# Long-Term Behavioral Programming Induced by Peripuberty Stress in Rats Is Accompanied by GABAergic-Related Alterations in the Amygdala

**DOI:** 10.1371/journal.pone.0094666

**Published:** 2014-04-15

**Authors:** Stamatina Tzanoulinou, Clara García-Mompó, Esther Castillo-Gómez, Vandana Veenit, Juan Nacher, Carmen Sandi

**Affiliations:** 1 Laboratory of Behavioral Genetics, Brain Mind Institute, School of Life Sciences, Ecole Polytechnique Fédérale de Lausanne, Lausanne, Switzerland; 2 Neurobiology Unit and Program in Basic and Applied Neurosciences, Cell Biology Department, Universitat de València, Valencia, Spain; 3 CIBERSAM: Spanish National Network for Research in Mental Health, Madrid, Spain; 4 Fundacion Investigacion Hospital Clinico de Valencia, INCLIVA, Valencia, Spain; Max Planck Institute of Psychiatry, Germany

## Abstract

Stress during childhood and adolescence is a risk factor for psychopathology. Alterations in γ-aminobutyric acid (GABA), the main inhibitory neurotransmitter in the brain, have been found following stress exposure and fear experiences and are often implicated in anxiety and mood disorders. Abnormal amygdala functioning has also been detected following stress exposure and is also implicated in anxiety and social disorders. However, the amygdala is not a unitary structure; it includes several nuclei with different functions and little is known on the potential differences the impact of early life stress may have on this system within different amygdaloid nuclei. We aimed here to evaluate potential regional differences in the expression of GABAergic-related markers across several amygdaloid nuclei in adult rats subjected to a peripuberty stress protocol that leads to enhanced basal amygdala activity and psychopathological behaviors. More specifically, we investigated the protein expression levels of glutamic acid decarboxylase (GAD; the principal synthesizing enzyme of GABA) and of GABA-A receptor subunits α2 and α3. We found reduced GAD and GABA-A α3, but not α2, subunit protein levels throughout all the amygdala nuclei examined (lateral, basolateral, basomedial, medial and central) and increased anxiety-like behaviors and reduced sociability in peripubertally stressed animals. Our results identify an enduring inhibition of the GABAergic system across the amygdala following exposure to early adversity. They also highlight the suitability of the peripuberty stress model to investigate the link between treatments targeting the dysfunctional GABAergic system in specific amygdala nuclei and recovery of specific stress-induced behavioral dysfunctions.

## Introduction

Exposure to stress during early life is a known risk factor for the development of mental illness [1]–[3]. In particular, childhood and adolescence are periods of high vulnerability to long-lasting programming of psychopathologies –including anxiety disorders– by stress [4], as reported in a variety of species, including humans [2], [5], [6], non-human primates [7], [8], and rodents [9]–[11]. Nevertheless, the neurobiological mechanisms translating early life stress effects into pathological anxiety are not sufficiently understood.

Neurocircuitry models of stress-related anxiety disorders implicate abnormalities in the functioning and connectivity of different brain regions, including the amygdala, hippocampus and medial prefrontal cortex, with amygdala dysfunction playing a prominent role [12]–[14]. The amygdala has been implicated in the processing of salience, particularly in response to fear stimuli [15], [16]. Enhanced amygdala reactivity can be detected in individuals with high state and trait anxiety levels exposed to emotionally arousing stimuli (for a review, see [17]) and has emerged as a hallmark in a variety of anxiety disorders, including post-traumatic stress disorder (PTSD) and social phobia [18], [19]. Importantly, neuroimaging studies in PTSD patients indicate that increased amygdala activation is not only observed following exposure to disorder-relevant stimuli [20]–[22], but also at rest [23] and during the completion of non-emotional tasks [24], [25].

Gamma-aminobutyric acid (GABA), the main inhibitory neurotransmitter in the brain, is one of the key messengers implicated in anxiety and mood disorders [26], [27]. Although scarce, available evidence suggests that differences in the underlying neurochemistry, particularly in the GABAergic system, might be involved in the differential amygdala responsiveness in stress-related anxiety disorders [28], [29]. GABA is synthesized from glutamate by the enzyme Glutamic Acid Decarboxylase (GAD) [30] which is encoded by two different genes that yield two different GAD isoforms, GAD65 and GAD67 [30]–[32]. GAD has often been used as an indicator of GABAergic-related function [33]–[35]. Reductions in GAD65 and GAD67 have been shown in the brains of autistic patients [33] and in association with bipolar disorder and depression [34].While manipulating GABAergic transmission systemically is well-known to strongly modulate anxiety-like behaviors [36]–[39] and amygdala reactivity [40], [41], targeting GABAergic transmission directly in the rat amygdala was found to affect anxiety-like behaviors [42], social interactions [43] and responses in the sympathetic nervous system [44]. Rodent studies have also reported evidence that stress exposure during the juvenile period can lead to increased anxiety-like behavior in adulthood [9], [45] as well as alterations in the expression of GABA-A receptor subunits in the amygdala [45], [46].

GABA-A receptors have been implicated in the modulation of anxiety in animals [43], [47] and in depression and anxiety-related disorders in humans [48]–[51]. The majority of GABA-A receptors in the central nervous system are heteropentamers which are generally composed of two α, two β and one γ subunit [52]–[54], with the specific composition of α subunits playing an important role in determining the functional and pharmacological properties of the receptor [55]–[57]. Substantial attention has been drawn to the α2 and α3 subunits as the selective activation of receptors containing these subunits was found to attenuate anxiety without having adverse sedation and addiction effects observed upon activation of α1-containing GABA-A receptors [53]. However, the quest for understanding the specific involvement of each of these subunits in modulating anxiety is still ongoing [54]. Moreover, preliminary evidence indicated that polymorphisms in the gene encoding for GABA-A α2 subunits (GABRA2) interact with early-life adversity to increase the risk for developing posttraumatic stress disorder [58].

The amygdala is not a unitary structure, but includes several nuclei with different functions [59], [60]. Thus, whereas the existing evidence implicates alterations in the amygdala GABAergic system in stress-related anxiety disorders, little is known about potential differences in the impact of early life stress in this system in different amygdala nuclei. Given the still limited resolution of neuroimaging approaches in humans, animal models are particularly well-suited to address this question. To this end, we aimed here at evaluating potential regional differences in the expression of essential elements of the GABAergic system: glutamic acid decarboxylase [GAD, the principal synthesizing enzyme of GABA [32]] and GABA-A receptor subunits α2 and α3, across several amygdala nuclei (lateral, basolateral, basomedial, medial and central) in adult rats subjected to a peripuberty stress (PPS) protocol that leads to altered anxiety-like behaviors and amygdala function [9]. The PPS protocol consisted of exposing rats to fearful experiences on 7 specific days, across postnatal days 28 to 42 (P28–42), to cover the equivalent of “childhood” (P28–P30) and puberty (P34, P36, P40 and P42) periods in the rat. PPS rats were shown to display enhanced activity in the medial and central, but not basolateral or lateral, amygdala nuclei following a social challenge, while displaying an overall increase in activity throughout the different amygdala nuclei examined under basal conditions [9]. Therefore, we hypothesized that peripuberty stress would lead to a reduction in the general GABAergic marker, GAD, throughout the amygdala nuclei. Moreover, given the involvement of both GABA-A α2 and α3 subunits in anxiety-related behavior [53] and that changes in these subunits have been reported in the context of early-life stress [45], [46], we hypothesized that PPS would lead to alterations in the α2 and/or α3 subunit in the amygdala of rats. Finally, we sought to further confirm dysfunctional anxiety-like behaviors and social interactions in this model.

## Materials and Methods

### Animals

The experimental subjects were the offspring of Wistar Han rats (Charles River Laboratories, France), bred in our animal facility. At weaning, at postnatal day 21 (P21), male rats from different litters were distributed among the different home cages by placing equivalent numbers of animals from each litter into either the stress or control group and the placement of siblings within the same home cage was avoided. Animals were housed 3 per standard plastic cage and kept on a 12 h light–dark cycle (lights on at 07h00 AM). Food and water were available *ad libitum*. All procedures were conducted in conformity with the Swiss National Institutional Guidelines on Animal Experimentation and approved by a license from the Swiss Cantonal Veterinary Office Committee for Animal Experimentation.

### Experimental Design

**Figure 1:**
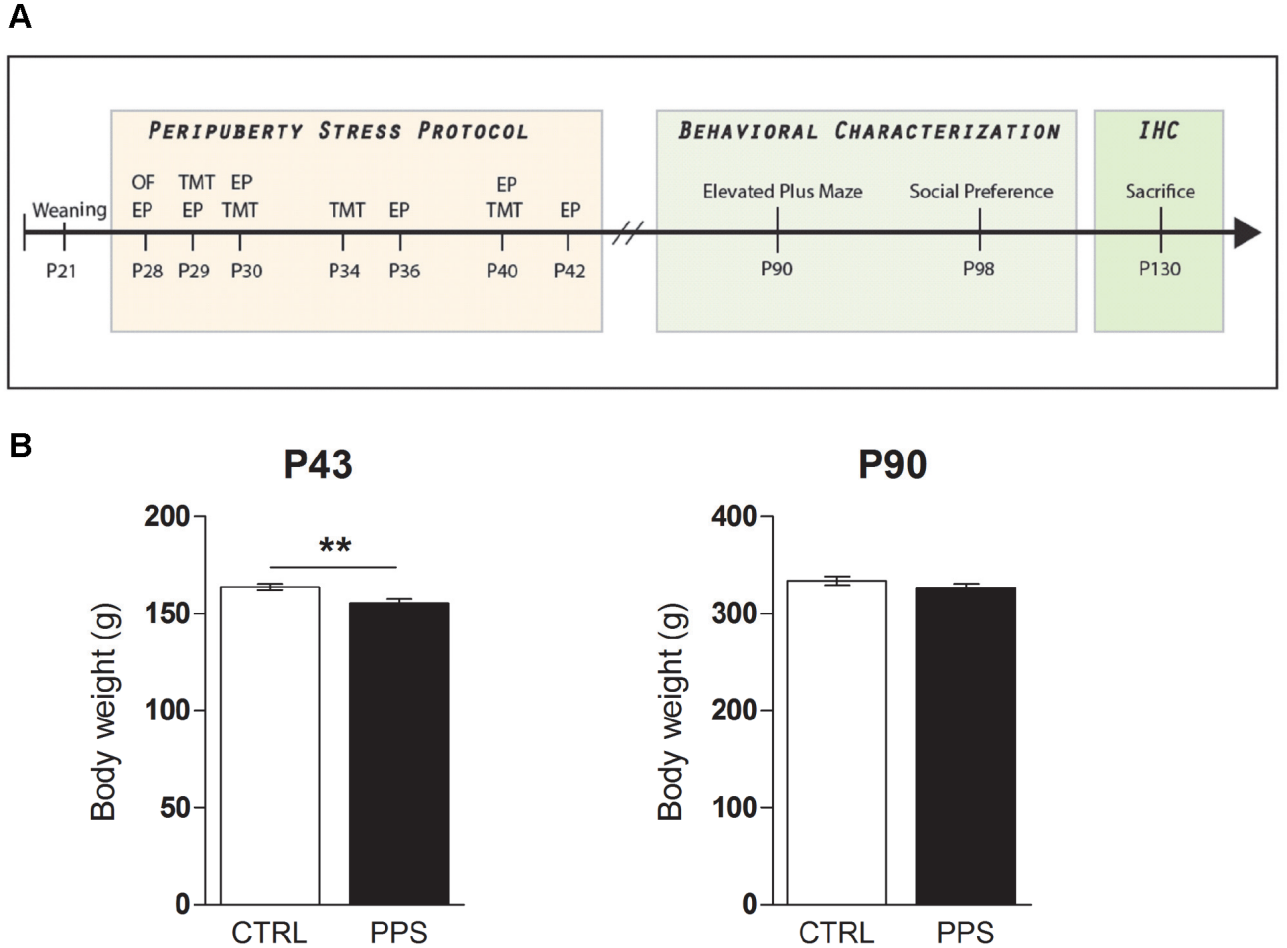
Experimental design and weight changes following peripuberty stress. Experimental design (A). All animals were weaned at P21 and at P28 they were assigned randomly to either CTRL or PPS groups. The peripuberty stress protocol consisted of exposure to an open field (OF) on the first day and then to elevated platform (EP) and predator odor (TMT), stressors that were presented in a variable manner until P42. The behavioral testing started at P90. Weight measurements were taken during the early-life period and at adulthood. IHC: Immunohistochemistry. Weight changes immediately after exposure to peripuberty stress and at adulthood (B). A reduction of body weight in PPS animals is evident one day after the end of the stress protocol, at P43, while no difference was observed at P90. ** p<0.01 versus CTRL; results are the mean ± SEM.

After weaning at P21, animals were randomly assigned to control (CTRL) or stress (PPS) groups and at P28 the stress protocol procedure began (Figure 1A). The behavioral characterization started from postnatal day P90, and was performed in 36 unstressed CTRL and 31 PPS animals. Following 3 consecutive days when all animals were handled, they were respectively tested for anxiety-like behaviors and sociability in the elevated plus maze and, one week later, in the social preference test. Four weeks later, a random subset of animals from each group was perfused and their brains taken for subsequent immunohistochemical brain analyses. Seven CTRL and 6 PPS animals were analyzed for GAD and GABA-A receptor subunit expression.

### Peripuberty Stress Protocol

The protocol of stress during puberty (PPS) is based on exposure to fear-induction procedures and was described in detail previously [9]. Briefly, following exposure to an open field for 5 minutes on P28, the stress protocol consisted of presenting two different fear-inducing stressors: the synthetic fox odor trimethylthiazoline (TMT) (9 ml) (Phero Tech Inc., Delta, BC, Canada), which was administered in a plastic box (38×27.5×31 cm) and exposure to an elevated platform (EP) (12×12 cm). Each stressor lasted 25 minutes. Following each stress session, the animals were returned to their home cages where, for the first 15 minutes, a transparent Plexiglas wall with holes separated each animal. The stressors were applied during the peripuberty period (a total of 7 days across postnatal day P28 to P42, i.e., on P28–P30, P34, P36, P40 and P42), during the light phase and followed a variable schedule. The control animals were handled on the days that their experimental counterparts were exposed to stress. At three time-points (P28, P34 and P43) body weight measurements were taken for both CTRL and PPS rats. Animals in the same cage were always assigned to the same experimental group (either CTRL or PPS).

### Elevated plus Maze

Anxiety-like behavior was assessed using the elevated plus maze (EPM) test [61] and the experiment was performed as previously reported [62]. The maze consists of two opposing open arms (50×10 cm) perpendicular to two enclosed arms (50×10×50 cm) that extend from a central platform (10×10 cm) elevated 65 cm above the floor. The rats were placed individually on the central platform facing a closed arm and allowed to explore the maze for 5 minutes. Their behavior was monitored using a video camera and analyzed with a computerized tracking system (Ethovision 3.1, Noldus IT). The times spent in the center zone, open and closed arms, as well as transitioning between different arms were recorded automatically. Additionally, the time spent head-dipping, stretching, self-grooming and rearing was manually scored online with the integrated module for manual scoring in Ethovision. The apparatus was cleaned with 1% acetic acid solution and dried thoroughly between each animal.

### Social Preference Test

The social preference test was adapted from the protocol described by Crawley and collaborators to investigate social affiliation in male mice [63] and followed the same conditions previously described [9], [62]. The apparatus used was a rectangular, three-chambered grey opaque polycarbonate box, consisting of a center (20×35×35 cm) and left and right compartments of 30×35×35 cm each. Dividing walls had retractable doorways allowing access to each chamber. Left and right compartments contained a central Plexiglas cylinder (15 cm diameter), transparent and perforated with small holes, where either a social (unfamiliar juvenile rat of 30±2 days old) or a non-social stimulus (yellow plastic bottle) were placed. The cylinder permits visual, tactile, auditory and olfactory communication. The juvenile rats were first habituated to the three-chambered apparatus by placing them individually in the box within the Plexiglas cylinder for 10 minutes during the 3 consecutive days preceding the social test. On testing day, the experimental rat was first placed in the middle chamber and allowed to habituate for 5 minutes. The doorways into the two side chambers were closed during this habituation phase. After the habituation period, the unfamiliar juvenile was placed in one of the side chambers and the object in the other side. The location of the juvenile and the object in the left vs. right side chamber alternated in a counterbalanced manner. Next, both doors to the side chambers were carefully removed and the experimental rat was allowed to explore the entire apparatus for a 10-minute session. Each trial was video-recorded (MediaCruise, Canopus Co., Ltd., Kobe, Japan) and manually scored offline by an experimenter blind to animals’ experimental conditions. The time spent sniffing each cylinder was scored to evaluate the level of preference for the unfamiliar juvenile as compared to the object. The rats were considered to explore the object and the juvenile when they approached the cylinders with their nose oriented towards the cylinders’ contents at a distance less than approximately 2 cm. Additionally, time spent rearing and self-grooming was also scored. The entire apparatus was cleaned with 1% acetic acid solution and dried properly between each trial.

### Perfusion

Two days after behavioral testing, eleven animals from each condition were perfused as reported previously [64]. The rats were anesthetized with a lethal dose of pentobarbital (Esconarkon, Streuli Pharma AG, 150 mg/kg body weight, solution provided by the EPFL veterinarian) and transcardially perfused with phosphate buffered saline (PBS) followed by 4% paraformaldehyde (PFA) for fixation. Following fixation, the brains were removed, post-fixed in 4% PFA for two days and cryoprotected in 30% sucrose solution until they sank to the bottom of the tube.

### Immunohistochemistry

In order to analyze whether PPS induced changes on the neuropil expression of glutamic acid decarboxylase 65/67 (GAD-6), GABA-A α2 and α3, three subseries (50 µm thick sections) from each group of animals were processed “free floating” for immunohistochemistry using the avidin-biotin-peroxidase (ABC) method [65]. Sections were first incubated for 1 minute in an antigen unmasking solution (0.01 M citrate buffer, pH 6) at 100°C. After cooling the sections down to room temperature, they were incubated with 3% H_2_O_2_ in phosphate buffered saline (PBS) for 10 minutes to block endogenous peroxidase activity. They were then treated for 1 hour with 10% normal donkey serum (NDS) (Jackson ImmunoResearch Laboratories) in PBS with 0.2% Triton-X100 (Sigma-Aldrich) and incubated for 24 hours at room temperature in the proper primary antibody: anti-GAD6, generated in mouse, (DSHB, 1∶500), anti GABA-A α2, generated in rabbit, (Synaptic Systems, 1∶1000) or GABA-A α3, generated in rabbit (Synaptic Systems, 1∶1000) with PBS containing 0.2% Triton-X-100 and 5% NDS. On the second day, sections were incubated for 1 hour at 25°C with the biotinilated secondary antibody: donkey anti-mouse IgG (Jackson ImmunoResearch Laboratories, 1∶200), or donkey anti-rabbit IgG (Jackson ImmunoResearch Laboratories, 1∶200), followed by an avidin-biotin-peroxidase complex (ABC; Vector Laboratories) for 30 minutes in PBS. Color development was achieved by incubating with 3,3′-diaminobenzidine tetrahydrochloride (DAB; Sigma-Aldrich) and 0.033% H_2_O_2_ for 4 minutes. Finally, sections were mounted on slides, dried for one day at room temperature, dehydrated with ascending alcohols and rinsed in xylene. After this, sections were coverslipped using Eukitt mounting medium (PANREAC). All studied sections passed through all procedures simultaneously in order to minimize any difference from the immunohistochemical staining itself. To avoid any bias in the analysis, all slides were coded prior to analysis and remained so until the experiment was completed.

From each immunostaining, we analyzed neuropil immunoreactivity in five nuclei within the amygdaloid complex [the lateral amygdaloid nucleus (LA), the basolateral amygdaloid nucleus (BLA), the basomedial amygdaloid nucleus (BMA), the medial amygdaloid nucleus (MeA) and the central amygdaloid nucleus (CeA)] in brain sections corresponding to Bregma −2.56 mm, Interaural 6.44 mm coordinates, using the same protocol previously described by Varea et al. (2007) [66]. In brief, from each immunostaining section examined per animal with an Olympus CX41 microscope under bright-field illumination, homogeneously lighted and digitalized using a CCD camera, photographs of the different amygdala nuclei were taken at 20X magnification and mean grey levels from 5 randomly selected square areas (15×15 µm) per nuclei and animal were measured and converted to optical densities (OD) using Image J software (NIH).

### Statistical Analysis

The data were analyzed with independent samples t-tests and repeated measures analysis of variance (ANOVA) as appropriate. All results represent the mean ± the standard error of the mean (SEM) and the significance was set at p<0.05. For the t-tests, if Levene’s test for equality of variances was significant, equal variance was not assumed and the altered degrees of freedom were rounded to the nearest whole number. For the repeated measures ANOVA, if Mauchly’s test of sphericity was significant, sphericity was not assumed and the Greenhouse-Geisser adjustment was used, rounding the degrees of freedom to the nearest whole number. The results were analyzed using the statistical package SPSS versions 13.0 and 17.0 and the graphs were created using GraphPad Prism 5.

## Results

### Peripuberty Stress Leads to Reduced Body Weight at Puberty That Is Recovered at Adulthood

In order to evaluate the stressful nature of the stress protocol [67]–[69], body weight for CTRL and PPS animals was monitored during and following the PPS protocol as well as in adulthood, at P90. No body weight differences were observed on P28 and P34 between CTRL and PPS rats (data not shown). However, there was a significant decrease in the body weight of PPS animals in the aftermath of the stress protocol, as measured at P43 (Figure 1B; P43: *t*_65_ = 3.024, p<0.01), illustrating the stressful character of the stress procedure. At adulthood (P90), no difference in body weight was observed between CTRL and PPS animals (Figure 1B; *t*_65_ = 1.163, n.s.). This lack of difference in body weight at adulthood allowed us to investigate the long-term impact of PPS on animals’ behavior and neurobiological correlates in the absence of apparent long-term physical effects.

### Peripuberty Stress Leads to Increased Anxiety-like Behavior at Adulthood

**Figure 2:**
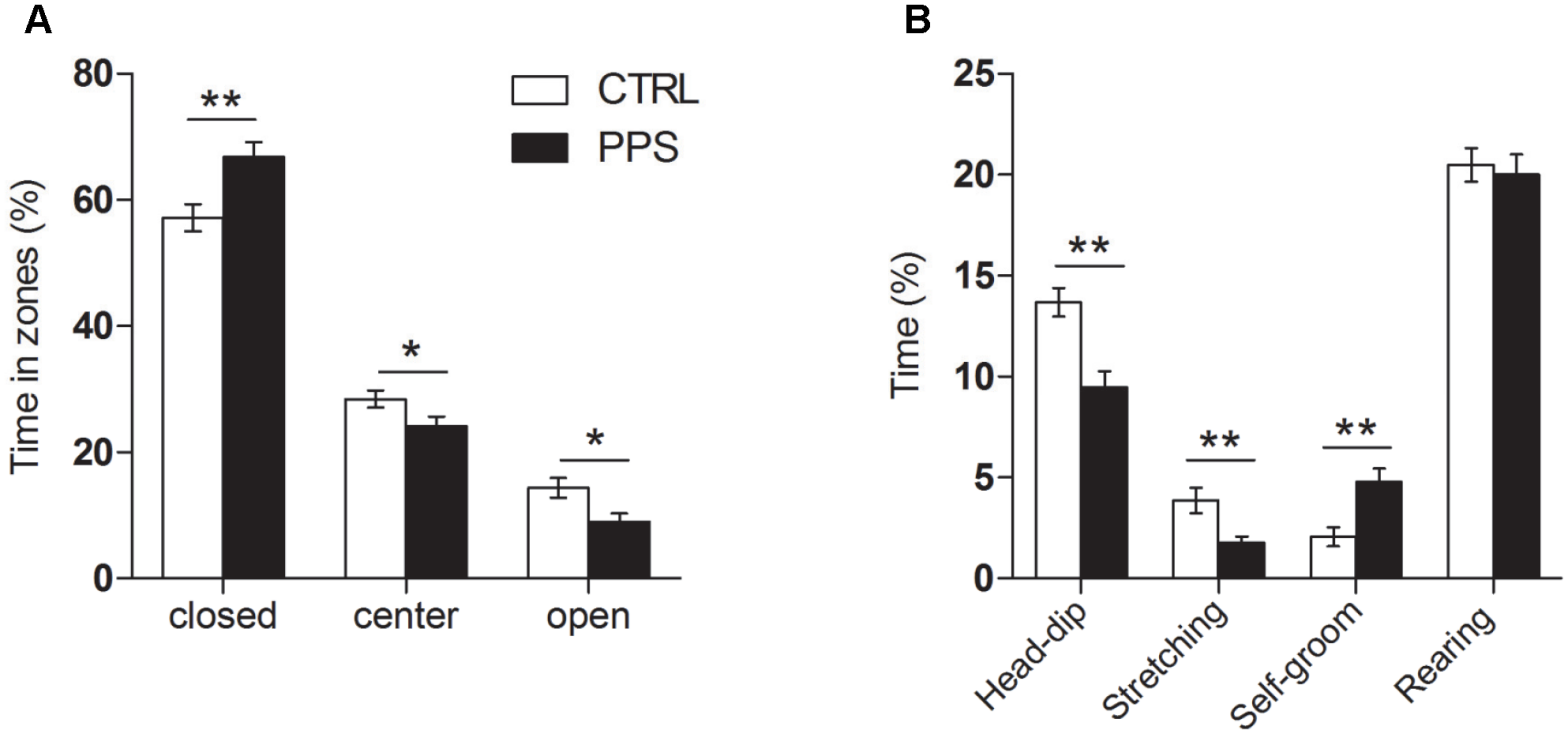
Anxiety-like behavior in the elevated plus maze. PPS animals spent more time in the closed arms of the maze, and less time in the center and open arms as compared to CTRL (A). In addition, they spent less time head-dipping and stretching and more time self-grooming than CTRL (B). * p<0.05, ** p<0.01 versus CTRL; results are the mean ± SEM.

Anxiety-like behavior was evaluated in the elevated plus maze. PPS animals spent more time in the closed arms and less time in the center and open arms of the maze as compared to CTRL rats (Figure 2A; closed arms: *t*_65_ = −2.999, p<0.01, center: *t*_65_ = 2.098 p<0.05, open arms: *t*_65_ = 2.591, p<0.05). Moreover, PPS animals exhibited reduced head-dipping (*t*_65_ = 3.955, p<0.01) and stretching (*t*_50_ = 3.034, p<0.01) behaviors, but increased self-grooming (*t*_56_ = −3.368, p<0.01), while no differences in rearing (*t*_65_ = 0.365, n.s.) were observed (Figure 2B).

### Peripuberty Stress Leads to Decreased Social Exploration at Adulthood

**Figure 3:**
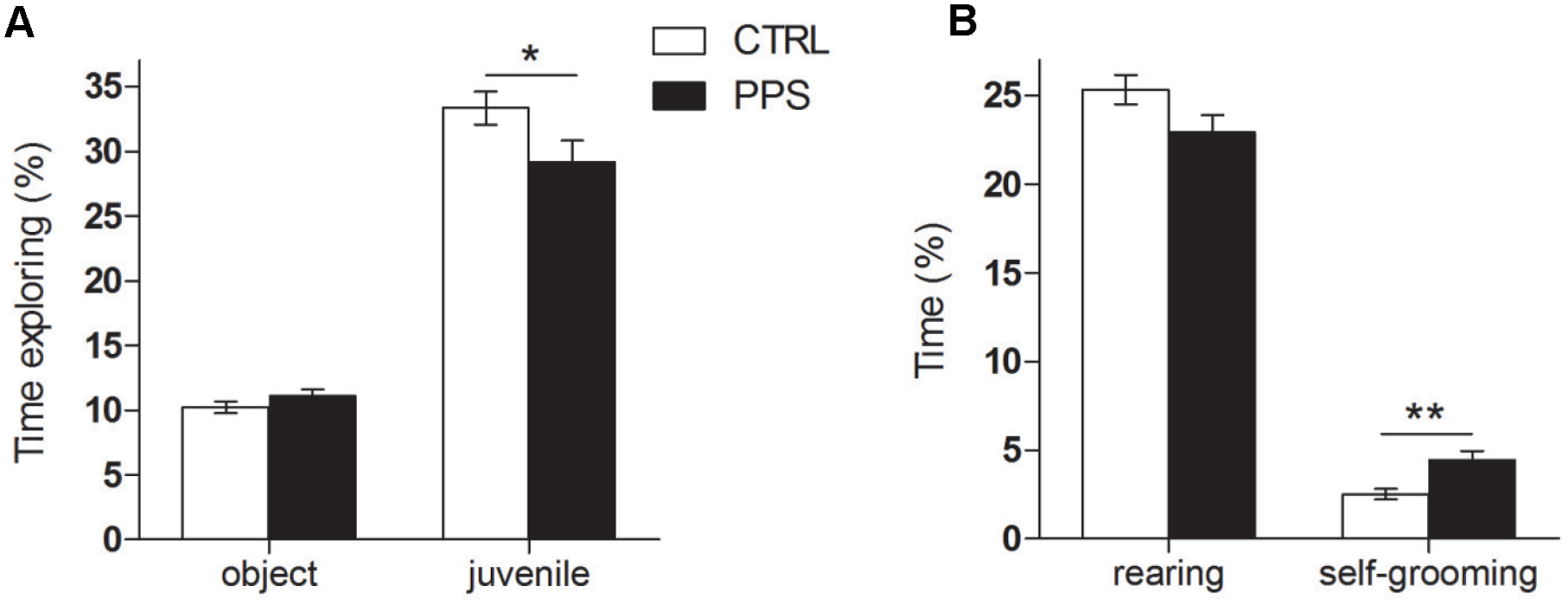
Sociability behavior in the social preference test. PPS animals spent comparable time exploring the object, but less time sniffing the juvenile rat as compared to CTRL (A). PPS animals exhibited more time self-grooming but comparable rearing behavior to the CTRL rats (B). * p<0.05, ** p<0.01 versus CTRL; results are the mean ± SEM.

The effects of PPS in sociability were evaluated with the social preference test. As expected, PPS animals spent less time exploring the conspecific juvenile than CTRL animals did (Figure 3A; *t*_65_ = 2.039, p<0.05). However, no differences between groups were found in the time spent exploring the inanimate object (Figure 3A; *t*_65_ = −1.330, n.s.). In addition, PPS animals did not show changes in rearing *(t*_65_ = 1.873, n.s.), but did display increased self-grooming when compared to CTRL (*t*_65_ = −3.413, p<0.01) (Figure 3B).

### Peripuberty Stress Results in Decreased Glutamic Acid Decarboxylase in the Amygdala

**Figure 4:**
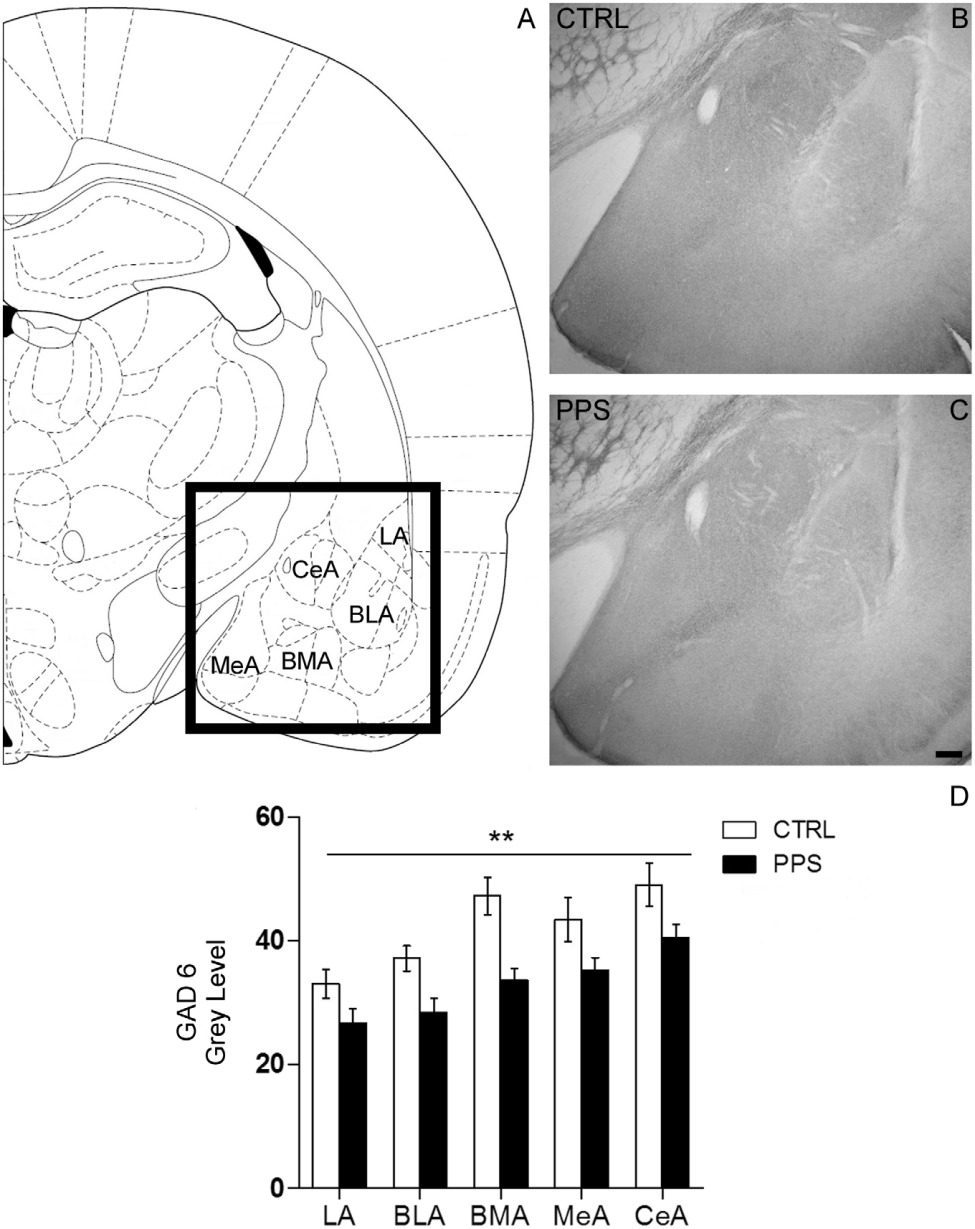
Effects of PPS on the expression of glutamic acid decarboxylase 65/67 (GAD6) in the amygdala of rats. (A) Schematic representation of the different amygdalar nuclei analyzed (Bregma −2.56 mm, Interaural 6.44 mm), modified from the Atlas of Paxinos and Watson (2006). (B & C) Photomicrographs showing the GAD6 neuropil inmunoreactivity in the amygdala of CTRL (B) and PPS (C) rats. Scale bar: 200 µm. Optical densitometry of neuropil immunoreactivity revealed a significant decrease of GAD protein levels in the amygdala of PPS animals as compared to CTRL (D). ** p<0.01 versus CTRL, results are the mean ± SEM.

Immunohistochemical analyses of the inhibitory marker GAD were performed in slices containing the amygdala of PPS and CTRL animals. GAD expression levels were quantified in different amygdala nuclei, including the lateral (LA), basolateral (BLA), basomedial (BMA), medial (MeA) and central (CeA) nuclei (see Figure 4A for the schematic localization of these nuclei and Figures 4B and 4C for representative immunostaining images from CTRL and PPS rats). As shown in Figure 4D, lower GAD levels across the different amygdala nuclei were found in PPS animals than in CTRL (*F*_1,11_ = 13.553, p<0.01), but no significant brain region×stress treatment interaction was found (*F*_2,21_ = 0.711, n.s). This reduction in GAD expression throughout the amygdala by PPS was confirmed in an independent cohort of animals (data not shown), while specificity was suggested by lack of changes observed in the cingulate cortex (Tzanoulinou et al., manuscript in preparation).

### Peripuberty Stress Results in Decreased Α3 GABA-A Receptor Subunit in the Amygdala without Affecting the Expression of the Α2 Subunit

**Figure 5:**
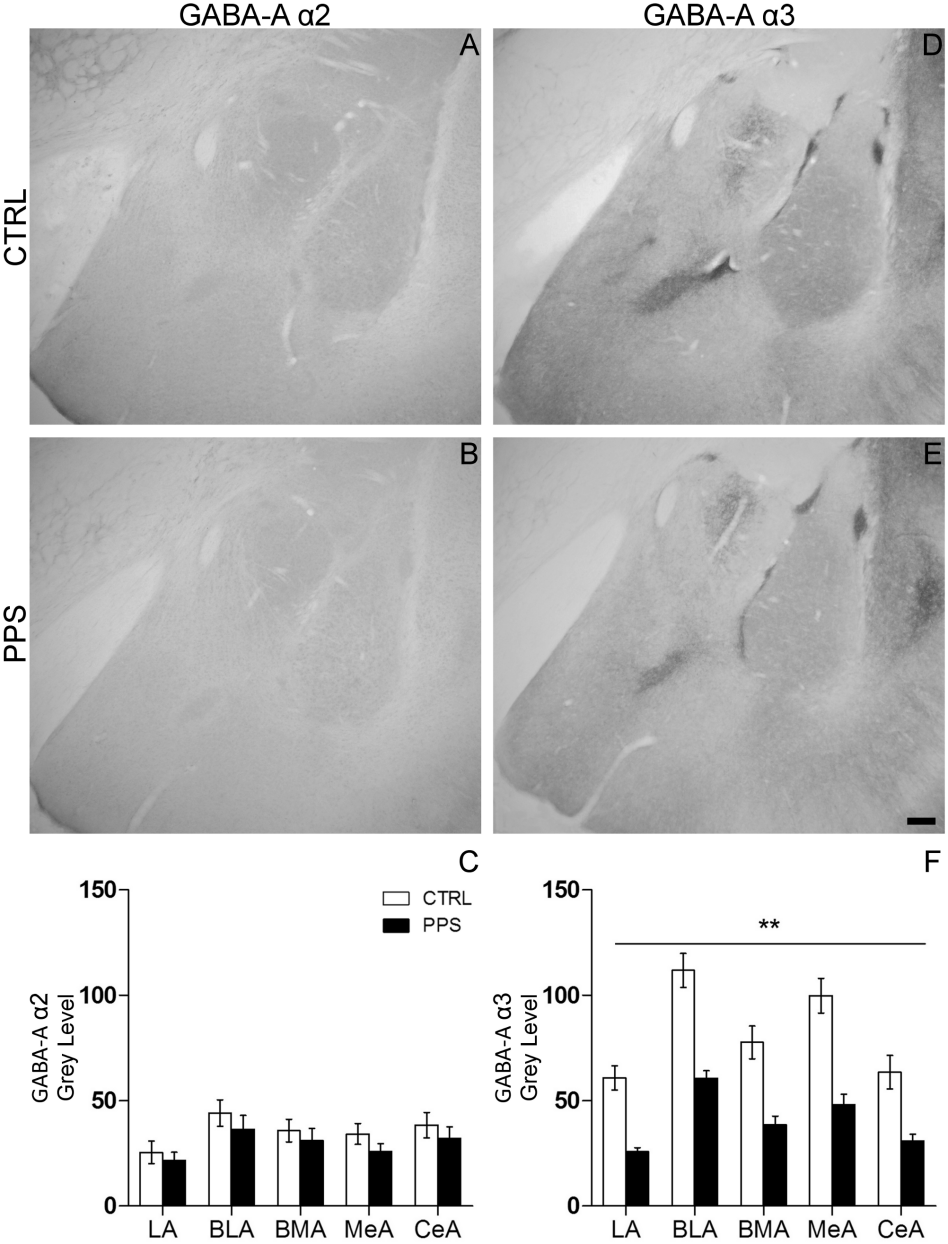
Effects of PPS on the expression of GABA-A receptor α2 subunit (GABA-A α2) and α3 subunit (GABA-A α3) in the amygdala of rats. (A & B) Photomicrographs showing the GABA-A α2 inmunoreactivity in the amygdala of CTRL (A) and PPS (B) rats. (D & E) Photomicrographs showing the GABA-A α3 inmunoreactivity in the amygdala of CTRL (D) and PPS (E) rats. Scale bar: 200 µm. Optical densitometry of neuropil immunoreactivity revealed no differences in the expression of GABA-A α2 protein levels between PPS and CTRL groups (C). However, a significant decrease of GABA-A α3 protein levels was observed in the amygdala of PPS as compared to CTRL rats (F). ** p<0.01 versus CTRL, results are the mean ± SEM.

The protein expression levels of the α2 and α3 GABA-A receptor subunits were examined using immunohistochemical analyses in the amygdala of CTRL and PPS animals. Figures 5A and 5B show representative immunostaining images of the GABA-A α2 subunit in the amygdala of CTRL and PPS rats. No difference was observed between CTRL and PPS rats (Figure 5C; *F*_1,11_ = 0.714, n.s.) nor a significant brain region x stress treatment interaction (*F*_4,44_ = 0.445, n.s). Figures 5D and 5E show representative immunostaining images of the GABA-A α3 subunit in the amygdala of CTRL and PPS animals. A reduction was observed across the amygdala in PPS rats as compared to CTRL (Figure 5F; *F*_1,11_ = 24.579, p<0.01). Moreover, there was a significant brain region x stress treatment interaction (*F*_4,44_ = 7.253, p<0.01) mainly due to a differential expression of the GABA-A α3 subunit across different amygdaloid nuclei. Therefore, our results show a significant reduction of the GABA-A α3 subunit across the amygdala nuclei of PPS rats, whereas no significant differences were observed between CTRL and PPS animals for the GABA-A α2 subunit.

## Discussion

Using a peripuberty stress protocol in rats that leads to increased activity throughout the amygdala at adulthood [9], we show here that protein levels of both the GABA marker, GAD, and the GABA-A α3, but not α2, subunit are reduced throughout all the amygdala nuclei examined (i.e., LA, BLA, BMA, MeA and CeA). We also show that these changes are present in the context of increased anxiety-like behaviors and reduced sociability displayed by PPS animals. These findings indicate a general susceptibility (as opposed to nucleus-specific vulnerability) across the amygdala to alterations in the long-term programming of the GABAergic system caused by exposure to stress around puberty. They also suggest that a deficient GABAergic system might underlie amygdala overactivity associated with anxiety and social dysfunctions triggered by early life stress.

We verified the increased anxiety-like responses and the reduced sociability previously reported for PPS animals [9]. In addition to spending less time in the open arms and center of the elevated plus maze, we found here that PPS animals show decreased head-dipping and stretching as well as other anxiety-characteristic behaviors, such as increased self-grooming. Self-grooming is known to increase with stress and to decrease upon anxiolytic treatment [70]–[72]. No differences were observed between PPS and CTRL animals in their general exploratory behavior. These observations are in line with previous studies in which rats subjected to stress during the pre-puberty period displayed increased anxiety-like behaviors at adulthood [11], [73]–[75], despite the fact that the protocol followed in those studies was shorter (essentially equivalent to the first 3 stress days in our protocol; i.e., from P27/28–P29/30) than the PPS protocol used in the current work (covering 4 additional days: P34, P36, P40, P42). However, the opposite pattern of results has been described when animals were tested during adolescence, in the aftermath of stress exposure [10], [11], [76], [77]. Social stress during adolescence in rats was also shown to decrease social interaction when assessed 3 weeks after the last stress experience [78].

The overall GAD reduction in the amygdala found in PPS animals strongly supports a long-term inhibitory programming of the GABAergic system in the amygdala by early life stress. GAD is responsible for synthesizing GABA [31] and is frequently used as a marker of GABAergic function [33]–[35]. In a previous study, we reported increased activation of the amygdala in PPS animals as measured by 2-Deoxy-Glucose (2-DG) uptake [9]. Given that activation levels measured by 2-DG can reflect both processes of inhibitory and excitatory neurotransmission, as both processes involve similar metabolic mechanisms [79], [80], our current results support a reduction in the inhibitory transmission –consequently leading to enhanced excitation in the amygdala of PPS animals– as an underlying mechanism. In fact, similar evidence supporting an enhanced excitation has been found after chronic stress in the amygdala of adult rodents. Chronic immobilization stress was previously found to induce dendritic hypertrophy in excitatory neurons in the BLA in rats [81] and atrophy in amygdaloid interneurons accompanied by significantly reduced levels of GAD67 mRNA and protein in mice [82]. Although more studies are needed to verify this view, a bias towards amygdala excitation in the excitation/inhibition balance would be in agreement with evidence in clinical populations that typically display alterations in emotionality and social behaviors, such as autism and anxiety disorders patients [83]–[85]. Interestingly, mice deficient in GAD65 show increased anxiety-like behaviors [86].

In addition, we show a persistent reduction of the α3, but not α2, GABA-A receptor subunit across the amygdala nuclei of peripubertally stressed animals. Whereas α2 GABA-A receptor subunits seem to be responsible for fast GABAergic postsynaptic currents, α3-containing GABA-A receptors in the amygdala have been implicated in tonic inhibition [87], [88]. Although considerable evidence implicates the α2 subunit in anxiety behavior [89], several lines of research have emphasized the involvement of the α3 subunit in mediating anxiety-related effects [90], [91]. In agreement with our findings, pharmacological evidence supports anxiogenic effects for reduced activation of α3-containing GABA-A receptors [91] while anxiolytic actions for its increased function [54], [90], [92]. Importantly, anxiolytic effects of an α3 subunit selective agonist were observed even in mice with benzodiazepine insensitive α2 GABA-A subunits [90]. On the other hand, juvenile stress (i.e., corresponding to the first part of the PPS protocol) was previously found to enhance the vulnerability of the amygdaloid GABAergic system following an emotional challenge at adulthood, with increases in both the α2 and α3 GABA-A subunits in the context of enhanced anxiety-like behavior [45], [46]. Although our results differ from these findings, there are important differences in the timing of stress and testing conditions [tissue harvesting for protein assessment was performed 24 hours following the emotional challenge in Jacobson-Pick et al. (2008) [46] and Jacobson-Pick and Richter-Levin (2012) [45], while several weeks following the last behavioral testing in our study] that could account for the discrepancy. Altogether, these findings illustrate the complexity of the link between the specific subunit composition of GABA-A receptors and in anxiety, whose understanding warrants further investigation.

Collectively, our findings are in line with the existing literature linking alterations of the GABAergic system with anxiety and mood disorders [26], [27], [93], [94]. They also fit with evidence in animals linking early adversity [95] and, more generally stress exposure [96] with alterations in the amygdala GABAergic system. Importantly, the modulation of GAD and GABA-A α3 levels was observed throughout the different amygdala nuclei. This is in contrast with our expectation to find modulations in specific amygdala nuclei [i.e., particularly in the central and medial amygdala where we had found significant activation following a resident-intruder test in PPS animals [9]], and with previous chronic stress studies performed at adulthood indicating opposite structural changes in different amygdala nuclei (i.e., reduced spine density in the MeA, while increased in the BLA) [97]. However, we should also note that GABAergic markers in the current study were measured under basal conditions (i.e., several weeks from the last behavioral challenge) and our previous imaging data in PPS animals under basal conditions had shown increased activity throughout the different amygdala nuclei [9]. Therefore, it is still possible that PPS animals might show a differential modulation of GAD and/or GABA-A receptor subunit levels when examined immediately after a challenge at adulthood. Furthermore, this lack of specificity for a particular amygdala nucleus in the observed reductions of GAD and α3 GABA-A subunit expression levels could be considered to mirror the broad spectrum of behavioral alterations that we have observed, so far, in the peripuberty stress model. In addition to the reduced social exploration and increased anxiety-like responses in the elevated plus maze described here, PPS animals were also found to display pathological aggression [9], [98] and impaired fear extinction [99], [100]. Existing evidence implicates the different amygdala nuclei in which we see a reduction of GABAergic markers in aspects of the dysfunctional behaviors observed in PPS animals which include the CeA in pathological aggression [101] and anxiety [43]; the lateral and basolateral amygdala in fear extinction [102], [103]; and the medial amygdala in social behaviors [104].

Our study does not address the mechanisms involved in the long-term programming of the amygdala GABAergic system by peripubertal stress and future studies should explicitly address this question. However, existing evidence points at the stress messengers corticosterone and the corticotropin releasing hormone (CRH) as potential candidates. We found that corticosterone injections given according to the same experimental schedule as stress administration in the PPS protocol recapitulate alterations induced by the PPS protocol in the social domain, though not in the elevated plus maze [62]. We also found evidence for the alteration of the CRH system in adult PPS animals and PPS-induced dysfunctions in the sociability and elevated plus maze tests to be reverted by treatment with a CRH receptor 1 antagonist during the post-stress period [105]. Previous studies showed that administration of either a CRH receptor agonist or corticosterone to BLA neurons *in vitro* or *in vivo* reduces local GABA-A inhibition while increasing the excitability of the network [106], [107]. Glucocorticoids are known to exert important programming effects [108], [109] and to interact with the GABAergic system [110]–[112]. Moreover, deletion of GABA-A α1 receptor subunit in CRH neurons was shown to lead to increased CRH expression in the amygdala and to increase anxiety-like behavior [113].

In summary, we present evidence for enduring reduction of GABAergic system-related markers across the amygdala following exposure to a peripuberty stress protocol in rats that also leads to a myriad of dysfunctional behaviors. Given evidence in the literature supporting a role for inhibited GABA neurotransmission in the amygdala in psychopathology, our study provides a valuable model to further investigate the link between treatments targeting the GABAergic system in specific amygdala nuclei and recovery of specific stress-induced behavioral dysfunctions.
